# Perioperative evaluation of elective surgical patients: is it possible to plan ICU admission?

**DOI:** 10.1186/cc11075

**Published:** 2012-03-20

**Authors:** LM Mozzoni, FR Ruggeri, MN Nastasi

**Affiliations:** 1 Ospedale Ceccarini Riccione, Italy

## Introduction

The aim of the study is to evaluate the possibility to predict ICU admission in elective surgical patients, studying the perioperative period variables.

## Methods

This is a prospective, nonintervention study concerning 207 patients, who have been operated on under elective conditions from January to October 2011. The group we studied was affected by thoracic (*n = *78) or abdominal (*n = *129) cancer. Mean age was 67.8 (SD 11.3; limits 24 to 91). ASA score III concerned 107 patients (51.7%) and score II 98 patients (47.3%). A senior anesthetist screened all patients before operation, assigning them to one of these three possible groups: G0 (patient who does not need ICU admission), G1 (patients who could need ICU admission), G2 (patients who definitely need ICU admission). Scheduling of patients into groups was made considering medical history, laboratory data, physical evaluation and type of surgery. Patients were studied from surgical intervention to discharge. All data were analyzed using IBM SPSS statistics v19 (SPSS Inc.), using adequate test and accepting *P *< 0.05.

## Results

Sixty-six patients (31.9% of all patients) were in G0, 70 (33.8%) in G1 and 71 (34.3%) in G2. The ASA score can distinguish patients in G0 and G2, but not in G1 (*P *< 0.05). The decision to schedule patients in a group arises mainly from the coexistence of both cardiovascular and respiratory diseases [1]. Ninety patients (43.5%) entered the ICU; 30 (42.8%) of these were in G1 and 34 (47.9%) in G2; 26 (39.4%) were in G0. Distribution in the three groups of ICU-admitted patients was similar (*P *= NS) and there was no significant relationship between the ASA score (and its distribution in the three groups) and ICU admission (*P *= NS). Patients admitted had undergone surgery of longer duration or had problems in the theater (low output syndrome, difficult weaning at the end of procedure, bleeding) or organizational problems (*P *< 0.05). ICU-admitted patients show a lower number of postoperative complications as arrythmias and wound infections (*P *< 0.05). Four patients died, all had been hospitalized in the ICU. The mortality rate was 1.9% (75% were in G2). Patients with complications requiring further surgery were 15 (7.2%), seven of which had been hospitalized in the ICU.

## Conclusion

Preoperative evaluation does not appear to be a significant predictor for ICU admission, which is determined by intraoperative or organizational factors. The ICU admission reduces the incidence of postoperative complications; mortality is mainly due to the immediate perioperative period.
